# Cemiplimab in Japanese patients with advanced non-small cell lung cancer

**DOI:** 10.1093/jjco/hyaf160

**Published:** 2025-10-29

**Authors:** Yuki Sato, Yoko Tani, Hidenobu Ishii, Seigo Katakura, Masahide Oki, Yasutaka Watanabe, Toshihide Yokoyama, Katsuhiko Naoki, Jean-Francois Pouliot, Manika Kaul, Anne Paccaly, Jennifer E Visich, Eric Kim, Jayakumar Mani, Yuntong Li, Israel Lowy, Frank Seebach, Melissa Mathias, Satoshi Ikeda

**Affiliations:** Department of Respiratory Medicine, Kobe City Medical Center General Hospital, Kobe, Japan; Department of Respiratory Medicine, Osaka Metropolitan University Hospital, Osaka, Japan; Division of Respirology, Neurology, and Rheumatology, Department of Internal Medicine, Kurume University Hospital, Kurume, Japan; Department of Thoracic Oncology, Kanagawa Cancer Center, Yokohama, Japan; Department of Respiratory Medicine, NHO Nagoya Medical Center, Nagoya, Japan; Division of Thoracic Oncology, Saitama Cancer Center, Saitama, Japan; Department of Respiratory Medicine, Kurashiki Central Hospital, Kurashiki, Japan; Department of Respiratory Medicine, Kitasato University Hospital, Kanagawa, Japan; Regeneron Pharmaceuticals, Inc., Tarrytown, NY, United States; Regeneron Pharmaceuticals, Inc., Tarrytown, NY, United States; Regeneron Pharmaceuticals, Inc., Tarrytown, NY, United States; Regeneron Pharmaceuticals, Inc., Tarrytown, NY, United States; Regeneron Pharmaceuticals, Inc., Tarrytown, NY, United States; Regeneron Pharmaceuticals, Inc., Tarrytown, NY, United States; Regeneron Pharmaceuticals, Inc., Tarrytown, NY, United States; Regeneron Pharmaceuticals, Inc., Tarrytown, NY, United States; Regeneron Pharmaceuticals, Inc., Tarrytown, NY, United States; Regeneron Pharmaceuticals, Inc., Tarrytown, NY, United States; Department of Respiratory Medicine, Kanagawa Cardiovascular and Respiratory Center, Yokohama, Japan; Department of Thoracic Oncology, Kansai Medical University, Osaka, Japan

**Keywords:** clinical trials, immunotherapy, immune checkpoint inhibitors, non-small cell lung cancer

## Abstract

**Background:**

EMPOWER-Lung 1 and EMPOWER-Lung 3 (phase 3 studies) demonstrated survival benefits for cemiplimab with/without chemotherapy in global (non-Japanese) patients with advanced non-small cell lung cancer (aNSCLC). This single-arm dose-expansion study assessed safety, tolerability, pharmacokinetics, and efficacy of first-line cemiplimab (350 mg intravenous every 3 weeks) as monotherapy/with chemotherapy in Japanese patients with aNSCLC.

**Methods:**

The primary objectives were safety, tolerability, and pharmacokinetics of cemiplimab as monotherapy/with chemotherapy. Secondary objectives included immunogenicity, tumor response (objective response rate [ORR], and duration of response [DOR]). Patients whose tumors expressed programmed cell death-ligand 1 (PD-L1) ≥50% on tumor cells received cemiplimab monotherapy (Cohort A; *n* = 60, safety; *n* = 50, efficacy). Patients whose tumors expressed any level of PD-L1 received cemiplimab plus four cycles of chemotherapy (Cohort C; *n* = 50).

**Results:**

Safety results were generally consistent with the known safety profile of cemiplimab. Treatment-emergent adverse events (grade ≥3) were experienced by 51.7% (31/60) in patients receiving cemiplimab monotherapy (Cohort A) and 68.0% (34/50) in those receiving cemiplimab + chemotherapy (Cohort C). Pharmacokinetic results were similar across both cohorts. In Cohort A patients with centrally confirmed PD-L1 ≥50%, ORR was 60.0% (30/50) with an observed DOR of 2.1–42.5 months. In Cohort C, ORR was 42.0% (21/50) with an observed DOR of 2.3–20.7 months. Immunogenicity was low in both cohorts.

**Conclusion:**

Cemiplimab demonstrated efficacy in Japanese patients as monotherapy for PD-L1 ≥50% and with chemotherapy irrespective of PD-L1 expression. Overall, cemiplimab demonstrated a favorable benefit–risk profile in Japanese patients.

## Introduction

Lung cancer remains the leading cancer diagnosis and cause of cancer deaths worldwide [1]. The prevalence of lung cancer in Japan is increasing [2], and population-based registries show that 80% of these diagnoses are of non-small cell lung cancer (NSCLC) [3]. Given that registry data show the 5-year survival rate for advanced NSCLC is <7% in Japan [4], there is an urgent need for treatments to improve prognosis in Japanese patients [5].

The current NSCLC treatment landscape has evolved to include the use of anti–programmed cell death-1 (PD-1) monoclonal antibodies in patients without actionable genetic alterations [6, 7]. Nivolumab was the first anti–PD-1 blocking antibody approved in Japan, for NSCLC in 2015 [8, 9]. In 2016, pembrolizumab monotherapy was approved for advanced NSCLC in the first-line setting in Japan [10].

Cemiplimab, a fully human monoclonal PD-1 antibody, is approved in the USA and Europe as monotherapy in adults for the first-line treatment of NSCLC expressing programmed cell death-ligand 1 (PD-L1) in ≥50% of tumor cells with no epidermal growth factor receptor (*EGFR*), anaplastic lymphoma kinase (*ALK*)*,* or ROS proto-oncogene 1 (*ROS1*) aberrations, where the patient has locally advanced disease but is not a candidate for definitive chemoradiation therapy, or has metastases. Cemiplimab is also approved in combination with platinum-based chemotherapy for the treatment of locally advanced NSCLC where the patient is not a candidate for surgical resection or definitive chemotherapy, or has metastatic NSCLC [11, 12]. These approvals in NSCLC were supported by the results from two phase 3 clinical trials of cemiplimab, EMPOWER-Lung 1 and EMPOWER-Lung 3 [11–16].

EMPOWER-Lung 1 (NCT03088540), a multicenter, open-label, randomized phase 3 trial, evaluated cemiplimab as monotherapy versus chemotherapy alone in patients with advanced NSCLC and PD-L1 expression in ≥50% of tumor cells [13, 15]. EMPOWER-Lung 3 (NCT03409614), a multicenter, double-blind, randomized phase 3 trial, evaluated cemiplimab in combination with chemotherapy versus chemotherapy alone in patients with advanced NSCLC and any level of PD-L1 expression [14, 16]. Primary analyses and updated results from the EMPOWER Lung-1 and EMPOWER-Lung 3 trials demonstrated significant survival benefits for cemiplimab with or without chemotherapy, with significant improvements in objective response rate (ORR), overall survival (OS), and progression-free survival (PFS) versus chemotherapy alone [13, 14]. These trials enrolled patients globally, including sites in Europe, South America, and Asia.

A two-part, open-label, phase 1 multicenter study (NCT03233139) was conducted to evaluate cemiplimab in Japanese patients. Part 1 evaluated the safety, tolerability, pharmacokinetics (PK), and clinical activity of two dosing regimens (250 mg or 350 mg as an intravenous [IV] infusion every 3 weeks [Q3W]) of cemiplimab monotherapy in Japanese patients with advanced malignancies [5].

Part 2 evaluated the safety, tolerability, PK, and efficacy of cemiplimab as monotherapy or in combination with chemotherapy for the first-line treatment of Japanese patients with advanced NSCLC. Here, we report the results for patients with PD-L1 ≥50% receiving cemiplimab monotherapy (Cohort A), and patients with any level of PD-L1 expression receiving cemiplimab plus chemotherapy (Cohort C) from Part 2 of this study.

## Methods

### Study design and patients

The methods and results for Part 1 of this study (NCT03233139) have been published previously [5].

**Figure 1:**
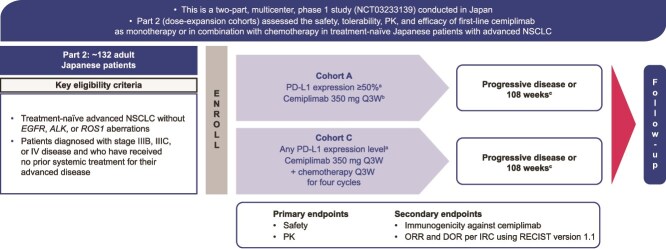
Study design. Flow diagram depicting Part 2 Cohort A and C. ^a^PD-L1 expression levels will be determined using the validated PD-L1 immunohistochemistry 22C3 pharmDx assay. ^b^At progression of disease, patients can receive four cycles of chemotherapy in combination with cemiplimab. ^c^Or early treatment discontinuation for another reason. *ALK,* anaplastic lymphoma kinase; DOR, duration of response; *EGFR*, epidermal growth factor receptor; IRC, independent review committee; NSCLC, non-small cell lung cancer; ORR, objective response rate; PD-L1, programmed cell death-ligand 1; PK, pharmacokinetics; Q3W, every 3 weeks; RECIST, Response Evaluation Criteria in Solid Tumors; *ROS1*, ROS proto-oncogene 1.

Part 2 is a single-arm, dose-expansion study in two cohorts of patients: first-line cemiplimab monotherapy (Cohort A) and first-line cemiplimab in combination with chemotherapy (Cohort C) in Japanese patients with advanced NSCLC (Fig. 1).

Eligible patients for inclusion in Part 2 (Cohorts A and C) of the study were aged ≥20 years, diagnosed with histologically or cytologically documented squamous or non-squamous NSCLC with stage IIIB, IIIC, or IV disease, and had received no prior systemic treatment for recurrent or metastatic NSCLC. Patients were required to have archival or newly obtained formalin-fixed tumor tissue from a metastatic/recurrent site which had not previously been irradiated, at least one radiographically measurable lesion per Response Evaluation Criteria in Solid Tumors version 1.1 (RECIST 1.1) criteria, an Eastern Cooperative Oncology Group (ECOG) performance status of ≤1, and a life expectancy of ≥3 months. Patients must have been born in Japan, and their biological parents and grandparents must all have been of Japanese origin. For Cohort A, the study population excluded patients who had smoked ≤100 cigarettes in their lifetime; Cohort C enrolled patients regardless of smoking history. Patients with *EGFR*, *ALK*, or *ROS1* aberrations were excluded from the study. Patients were also excluded if they had a history of interstitial lung disease (including active, non-infective pneumonitis), and had active, known, or suspected autoimmune disease that had required systemic treatment in the past 2 years. All patient samples were sent to a central laboratory for confirmatory testing of PD-L1 expression levels, which were determined by using the validated PD-L1 immunohistochemistry (IHC) 22C3 pharmDx assay (Agilent Technologies, Inc. [previously known as Dako]) (Fig. 1).

Patients with NSCLC with PD-L1 ≥50% were enrolled into Cohort A to receive cemiplimab monotherapy 350 mg Q3W by IV infusion for up to 108 weeks. In Cohort A, patients were enrolled based on local testing results of PD-L1 expression in ≥50% of tumor cells, and all patient samples were sent to a central laboratory for confirmatory testing of PD-L1 expression using the PD-L1 IHC 22C3 pharmDx assay. Cohort C enrolled patients with any PD-L1 status to receive cemiplimab at 350 mg Q3W (for 108 weeks) in combination with investigator’s choice of one of either paclitaxel or pemetrexed combined with carboplatin or cisplatin (option 1a: paclitaxel 200 mg/m^2^ IV plus carboplatin area under the curve (AUC) of 5 or 6 mg/ml/min IV, option 1b: paclitaxel 200 mg/m^2^ IV plus cisplatin 75 mg/m^2^ or option 2a: pemetrexed 500 mg/m^2^ IV plus carboplatin AUC of 5 or 6 mg/ml/min IV, option 2b: 500 mg/m^2^ IV plus cisplatin 75 mg/m^2^ IV) Q3W for four cycles (dosing cycles were 21 days). PD-L1 expression was not required for enrollment into Cohort C. The study consisted of a pretreatment screening period (~4 weeks), an on-study treatment period (~108 weeks), and a follow-up post-treatment period (~24 weeks).

### Study objectives

The primary objectives were to assess the safety, tolerability, and PK of cemiplimab as monotherapy or cemiplimab in combination with chemotherapy in Japanese patients with advanced NSCLC. Secondary objectives included immunogenicity of cemiplimab and tumor response (ORR and duration of response [DOR]) in patients with PD-L1 in ≥50% of tumor cells receiving (i) cemiplimab monotherapy as first-line treatment (Cohort A), and (ii) cemiplimab plus chemotherapy for the first-line treatment of patients whose tumors express any level of PD-L1 (Cohort C). Exploratory objectives were PFS, OS, and correlation between PD-L1 expression level at baseline with the efficacy of the study treatment.

The data cut-off date was 5 September 2023, for Cohort A, and 18 October 2023, for Cohort C. The database lock for both cohorts occurred on 8 December 2023.

### Assessments

#### Safety

The incidence and severity of treatment-emergent adverse events (TEAEs) was graded according to the National Cancer Institute Common Terminology Criteria for Adverse Events version 4.03 [17]. The study investigators assessed the relatedness of adverse events to study treatment. The safety analyses were conducted for the full analysis set, which included all patients who received cemiplimab.

#### Tumor response

Radiologic scans (computed tomography or magnetic resonance imaging) were performed at screening and then after every three cycles (one cycle = 21 days). Scans began at week 9 and continued until the end of the study (the on-treatment period, unless there was progressive disease), then were performed at follow-up visit 1 or 1–7 days from the last cycle visit, and every 56 days (at follow-up visits 2–4). ORR was assessed by an independent review committee (IRC) using RECIST 1.1, defined as the proportion of patients with a best response of confirmed complete response (CR) or partial response (PR). Among patients with a tumor response, DOR was defined as time between the date of first response (CR or PR) and the date of the first documented tumor progression or death due to any cause. For Cohort A, the efficacy analysis was conducted for patients who had confirmed PD-L1 ≥50% per central testing. For Cohort C, the efficacy analysis was conducted for the full analysis set.

#### Immunogenicity

Immunogenicity was assessed by measuring anti-cemiplimab antibodies (anti-drug antibodies [ADAs] and neutralizing ADAs) in serum from blood samples collected pre-dose throughout the treatment and follow-up periods. The ADA analysis included all patients who received any study drug and had at least one non-missing ADA result after the first dose of cemiplimab.

#### Pharmacokinetics

The PK analysis set included all patients who received the study drug and had at least one non-missing cemiplimab concentration result after the first dose until the end of the study.

Blood samples were collected pre-dose and at different time points throughout treatment and follow-up to determine cemiplimab PK, including trough concentration (C_trough_) and maximum concentration (C_max_). Functional cemiplimab concentrations were measured in serum using a validated enzyme-linked immunosorbent assay.

### Statistical analyses

This is an observational study and therefore no statistical hypothesis was tested. For Cohort A, it was estimated that a sample size of 50 patients would be sufficient to evaluate ORR as well as one-sided 95% confidence intervals (CIs) and precisions. For Cohort C, a sample size of 47 was required for hypothesis testing, with a 0.05 one-sided alpha and 80% power, although there was no hypothesis testing planned for this cohort. Approximately 50 patients would be sufficient to evaluate ORR with the one-sided 95% CIs and precisions.

The primary efficacy analysis was conducted when all evaluable patients who remained on study had the opportunity to be followed for at least three tumor assessments (i.e. ≥28 weeks [includes 27 weeks for three tumor assessments plus a 1-week assessment window]). For the tumor response analysis, ORR per IRC using RECIST 1.1 was summarized using descriptive statistics and 95% one-sided asymptotic CIs (95% one-sided: lower limit to 100%). The DOR per IRC was summarized using the Kaplan–Meier method.

## Results

### Baseline characteristics and clinical variables

Of a total of 153 patients screened, 110 were enrolled at ≤30 locations across Japan. At the data cut-offs, 60 patients with PD-L1 expression ≥50% on tumor cells based on local PD-L1 results were enrolled into Cohort A (cemiplimab monotherapy). The safety analyses included all patients who received cemiplimab (*n* = 60), and the primary efficacy analyses included 50 of these patients with PD-L1 expression in ≥50% of tumor cells confirmed by central laboratory PD-L1 testing. A total of 50 patients with any level of PD-L1 expression, all of whom were included in the safety and efficacy analyses, were enrolled into Cohort C (cemiplimab + chemotherapy).

Baseline demographic and clinical characteristics were generally similar across both cohorts. In Cohort A, the median age was 70 years, and the majority (80.0%) were male. Overall, 26.7% of patients had squamous histology, and 60.0% had an ECOG performance status of 1 (Table 1). Similarly, for patients in Cohort C, the median age was 65 years, 76.0% were male, 26.0% had squamous histology, and 70.0% had an ECOG performance status of 1 (Table 1). Most patients had a histology of adenocarcinoma: 65.0% in Cohort A; and 64.0% in Cohort C. Patients in Cohort C received one of three chemotherapy regimens in combination with cemiplimab: 18 patients (36.0%) received paclitaxel plus carboplatin, 23 patients (46.0%) received pemetrexed plus carboplatin, and 9 patients (18.0%) received pemetrexed plus cisplatin.

**Table 1:** Baseline demographic and clinical variables

	**Cemiplimab monotherapy** **(Cohort A)** **(*n* = 60)**	**Cemiplimab + chemotherapy** **(Cohort C)** **(*n* = 50)**
Age, years		
Median	70.0	65.0
Range	47.0–87.0	44.0–82.0
≥65, *n* (%)	46 (76.7)	26 (52.0)
Sex, *n* (%)		
Male	48 (80.0)	38 (76.0)
Female	12 (20.0)	12 (24.0)
ECOG performance status, *n* (%)		
0	24 (40.0)	15 (30.0)
1	36 (60.0)	35 (70.0)
Smoking status, *n* (%)		
Never smoked	0	7 (14.0)
Current smoker	8 (13.3)	15 (30.0)
Past smoker	52 (86.7)	28 (56.0)
PD-L1 expression, *n* (%)^a^		
≥50%	50 (83.3)	14 (28.0)
<50%	8 (13.4)	27 (54.0)
Missing	2 (3.3)	9 (18.0)
Histology, *n* (%)		
Adenocarcinoma	39 (65.0)	32 (64.0)
Squamous	16 (26.7)	13 (26.0)
Other	5 (8.3)	5 (10.0)

ECOG, Eastern Cooperative Oncology Group; PD-L1, programmed cell death-ligand 1.

^a^PD-L1 expression levels in Cohort A were based on central testing. PD-L1 expression was not required for enrollment in Cohort C, and results are based on available data from local or central testing.

### Treatment exposure and median duration of follow-up

As of the data cut-off, the mean duration of exposure for patients who received cemiplimab monotherapy (Cohort A) was 45.5 weeks (median [range] 29.1 [3.0–121.7] weeks). The median duration of follow-up was 12.0 months (Table 2). 

**Table 2:** Summary of safety results for cemiplimab monotherapy or cemiplimab in combination with chemotherapy

	**Cemiplimab monotherapy** **(Cohort A)** **(*n* = 60)**	**Cemiplimab + chemotherapy** **(Cohort C)** **(*n* = 50)**
Duration of treatment exposure, weeks		
Mean (SD)	45.5 (40.6)	31.8 (24.5)
Median (range)	29.1 (3.0–121.7)	26.7 (0.9–104.9)
Duration of follow-up, months, median (min:max)	12.0 (1.4:44.9)	9.0 (0.2:23.5)
TEAEs, *n* (%)		
Any	59 (98.3)	50 (100.0)
Serious	27 (45.0)	24 (48.0)
Grade ≥3	31 (51.7)	34 (68.0)
Leading to treatment discontinuation	22 (36.7)	19 (38.0)
Leading to death	0	2 (4.0)
Treatment-related TEAEs, *n* (%)		
Any	55 (91.7)	50 (100.0)
Grade ≥3	18 (30.0)	32 (64.0)
Immune-mediated TEAEs (sponsor-identified), *n* (%)		
Any	24 (40.0)	18 (36.0)
Grade ≥3	8 (13.3)	9 (18.0)
TEAEs occurring in ≥10% of patients in the cemiplimab monotherapy cohort (Cohort A) (*n* = 60), *n* (%)	**Any grade**	**Grade ≥3**
Infusion-related reaction	19 (31.7)	1 (1.7)
Pruritus	15 (25.0)	0
Decreased appetite	14 (23.3)	3 (5.0)
Pneumonitis	13 (21.7)	3 (5.0)
Constipation	12 (20.0)	1 (1.7)
Nausea	12 (20.0)	1 (1.7)
Rash	12 (20.0)	1 (1.7)
Dry skin	10 (16.7)	1 (1.7)
Insomnia	10 (16.7)	1 (1.7)
Pyrexia	10 (16.7)	0
Hypothyroidism	9 (15.0)	0
Malaise	9 (15.0)	0
Arthralgia	8 (13.3)	0
Diarrhea	8 (13.3)	2 (3.3)
Stomatitis	8 (13.3)	0
Vomiting	8 (13.3)	1 (1.7)
Alanine aminotransferase increased	7 (11.7)	2 (3.3)
Back pain	7 (11.7)	0
Headache	7 (11.7)	0
Edema peripheral	7 (11.7)	0
Pneumonia	7 (11.7)	1 (1.7)
Eczema	6 (10.0)	0
Rash maculo-papular	6 (10.0)	3 (5.0)
TEAEs occurring in ≥10% of patients in the cemiplimab + chemotherapy cohort (Cohort C) (*n* = 50), *n* (%)	**Any grade**	**Grade ≥3**
Constipation	24 (48.0)	0
Anemia	21 (42.0)	6 (12.0)
Decreased appetite	15 (30.0)	3 (6.0)
Nausea	15 (30.0)	2 (4.0)
Neutrophil count decreased	13 (26.0)	10 (20.0)
Alopecia	11 (22.0)	0
Malaise	10 (20.0)	0
Rash	9 (18.0)	0
Arthralgia	9 (18.0)	0
Platelet count decreased	9 (18.0)	2 (4.0)
Diarrhea	8 (16.0)	2 (4.0)
Stomatitis	8 (16.0)	0
Alanine aminotransferase increased	8 (16.0)	3 (6.0)
Peripheral sensory neuropathy	8 (16.0)	0
Edema peripheral	7 (14.0)	0
Hyponatremia	7 (14.0)	2 (4.0)
Infusion-related reaction	6 (12.0)	2 (4.0)
Pruritus	6 (12.0)	1 (2.0)
Insomnia	6 (12.0)	0
Pyrexia	6 (12.0)	1 (2.0)
Pneumonia	6 (12.0)	3 (6.0)
Rash maculo-papular	6 (12.0)	0
Aspartate aminotransferase increased	6 (12.0)	2 (4.0)
White blood cell count decreased	6 (12.0)	3 (6.0)
Pneumonitis	5 (10.0)	1 (2.0)
Vomiting	5 (10.0)	0

SD, standard deviation; TEAE, treatment-emergent adverse event.

TEAEs (≥10% of patients) were reported. Adverse events were graded according to the National Cancer Institute Common Terminology Criteria for Adverse Events version 4.03.

As of data cut-off, the mean duration of exposure for cemiplimab in combination with chemotherapy (Cohort C) was 31.8 weeks (median [range] 26.7 [0.9–104.9] weeks). The median duration of follow-up was 9.0 months (Table 2).

### Safety

TEAEs of any grade were experienced by 98.3% of patients treated with cemiplimab monotherapy (Cohort A [safety analysis set, *n* = 60]), and by 100.0% of patients treated with cemiplimab in combination with chemotherapy (Cohort C [safety analysis set, *n* = 50]; Table 2).

In Cohort A, 51.7% and 45.0% of patients experienced at least one grade ≥3 TEAE and at least one serious TEAE, respectively. Overall, 36.7% of patients discontinued treatment due to a TEAE. There were no TEAEs resulting in death (Table 2). The most common TEAEs of all grades (experienced by ≥20% of patients) included infusion-related reaction (31.7%), pruritus (25.0%), decreased appetite (23.3%), and pneumonitis (21.7%), followed by rash, nausea, and constipation (20.0% each) (Table 2). The most common grade ≥3 TEAEs (experienced by ≥5% of patients) were maculopapular rash, pneumonitis, and decreased appetite (5.0% each). Overall, in Cohort A, 91.7% of patients experienced at least one treatment-related TEAE, including 30.0% of patients who experienced at least one grade ≥3 treatment-related TEAE (Supplementary Table 1).

In Cohort C, 68.0% and 48.0% of patients experienced at least one grade ≥3 TEAE and at least one serious TEAE, respectively. Overall, 38.0% of patients discontinued treatment due to a TEAE. There were two deaths resulting from TEAEs: one was due to grade 5 immune-mediated pneumonitis during the post-treatment period; and the second was due to cerebellar hemorrhage during the on-treatment period that was determined to be unrelated to the study drug (Table 2). The most common TEAEs experienced by ≥20% of patients included constipation (48.0%), anemia (42.0%), decreased appetite (30.0%), nausea (30.0%), decreased neutrophil count (26.0%), alopecia (22.0%), and malaise (20.0%) (Table 2). The most common grade ≥3 TEAEs (≥5% of patients) were decreased neutrophil count (20.0%); anemia (12.0%); and increased alanine aminotransferase, decreased white blood cell (WBC) count, decreased appetite, neutropenia, and pneumonia (6.0% each). All patients (100.0%) experienced at least one treatment-related TEAE, including 64.0% of patients who experienced at least one grade ≥3 treatment-related TEAE (Supplementary Table 1). Immune-related TEAEs (sponsor-identified) for Cohorts A and C are shown in Supplementary Table 2.

No new safety signals were identified in either cohort. Overall, safety in Japanese patients was comparable to the global population.

### Pharmacokinetics

Overall, PK results were similar across both cohorts, demonstrating no effect of chemotherapy on cemiplimab exposure. In Cohort A (cemiplimab monotherapy, *n = 60*) the mean baseline body weight was 58.8 kg. Following the first dose of cemiplimab, the mean (standard deviation [SD]) C_trough_ was 26.6 (7.67) mg/L in 53 patients and the mean (SD) C_max_ was 145 (37.8) mg/L in 60 patients. At the steady state (assessed on week 21 of treatment or cycle 7 day 1), mean (SD) C_trough_ was 67.8 (23.5) mg/L in 35 patients and C_max_ was 193 (36.9) mg/L in 35 patients. Overall, steady-state mean cemiplimab exposure was shown to have increased by 2.5-fold for C_trough_ and 1.3-fold for C_max_ relative to the first dose of cemiplimab (Table 3).

**Table 3:** Summary of PK results for cemiplimab monotherapy and cemiplimab in combination with chemotherapy

	**Cemiplimab monotherapy** **(Cohort A)** **(*n* = 60)**	**Cemiplimab + chemotherapy** **(Cohort C)** **(*n* = 50)**
Mean baseline body weight, kg, (min:max)	58.82 (35.6:92.2)	60.00 (43.1:106.4)
After the first dose		
C_trough_ (mg/L)	Cohort A (*n =* 53)	Cohort C (*n =* 43)
Mean (SD)	26.6 (7.67)	25.1 (14.3)
Median (IQR)	25.4 (21.9–30.7)	22.9 (16.4–30.7)
C_max_ (mg/L)	Cohort A (*n =* 60)	Cohort C (*n =* 50)
Mean (SD)	145 (37.8)	144 (36.9)
Median (IQR)	137 (124–159)	132 (115–162)
At steady state		
C_trough_ (mg/L)	Cohort A (*n =* 35)	Cohort C (*n =* 27)
Mean (SD)	67.8 (23.5)	81.6 (45.1)
Median (IQR)	69.3 (53.8–80.8)	70.6 (53.1–105)
C_max_ (mg/L)	Cohort A (*n =* 35)	Cohort C (*n =* 27)
Mean (SD)	193 (36.9)	181 (40.8)
Median (IQR)	186 (159–215)	180 (150–211)

C_max_, maximum concentration; C_trough_, trough concentration; IQR, interquartile range; SD, standard deviation.

In Cohort C (cemiplimab + chemotherapy, *n* = 50), the mean baseline body weight was 60.0 kg. Following the first dose of cemiplimab, the mean (SD) C_trough_ was 25.1 (14.3) mg/L in 43 patients and C_max_ was 144 (36.9) mg/L in 50 patients. At steady state, C_trough_ and C_max_ were 81.6 (45.1) mg/L and 181 (40.8) mg/L in 27 patients and 27 patients, respectively; steady state was assessed on week 21 of treatment or cycle 7 day 1. The mean steady-state cemiplimab exposure was increased by 3.3-fold for C_trough_ and 1.3-fold for C_max_ compared with the first dose of cemiplimab (Table 3).

### Efficacy

In Cohort A (cemiplimab monotherapy), among patients with confirmed PD-L1 ≥50% by central PD-L1 testing (*n* = 50), the overall ORR was 60% per IRC (90% CI: 48.6–71.4%) with two CRs and 28 PRs. The observed DOR ranged from 2.1 to 42.5 months (Table 4). The majority of responding patients (86.7%) had an observed DOR >6 months. The Kaplan–Meier median DOR per IRC was not reached (95% CI: 19.2 months–not evaluable [NE]).

**Table 4:** Summary of efficacy results for cemiplimab monotherapy and cemiplimab in combination with chemotherapy

	**Cemiplimab monotherapy** **(Cohort A)** **(*n* = 50)**^**a**^	**Cemiplimab + chemotherapy** **(Cohort C)** **(*n* = 50)**
Complete response, *n* (%)	2 (4.0)	2 (4.0)
Partial response, *n* (%)	28 (56.0)	19 (38.0)
ORR, *n* (%)	30 (60.0)	21 (42.0)
90% CI, %	48.6–71.4	30.5–53.5
ORR by PD-L1 expression, *n* (%)		
≥90%	18/29 (62.1)	–
>60 to <90%	8/13 (61.5)	–
≥50 to ≤60%	4/8 (50.0)	–
≥50%	–	5/14 (35.7)
1–49%	–	7/16 (43.8)
<1%	–	6/11 (54.5)
DOR		
Kaplan–Meier median, months (95% CI)	NR (19.2–NE)	NR (6.6–NE)
Range, months	2.1–42.5	2.3–20.7
≥6 months, *n* (%)	26/30 (86.7)	16/21 (76.2)
≥12 months, *n* (%)	15/30 (50.0)	6/21 (28.6)

CI, confidence interval; DOR, duration of response; NE, not evaluable; NR, not reached; ORR, objective response rate; PD-L1, programmed cell death-ligand 1.

^a^As described in Methods, the primary efficacy analysis in Cohort A was conducted for 50 patients with centrally confirmed PD-L1 expression in ≥50% of tumor cells.

In Cohort A, response rates improved with higher PD-L1 (≥50%) levels (Table 4). Patients with PD-L1 ≥90% (*n* = 29) had an ORR of 62.1%, those with PD-L1 >60% to <90% (*n* = 13) had an ORR of 61.5%, and those with PD-L1 ≥50 to ≤60% (*n* = 8) had an ORR of 50%.

**Figure 2:**
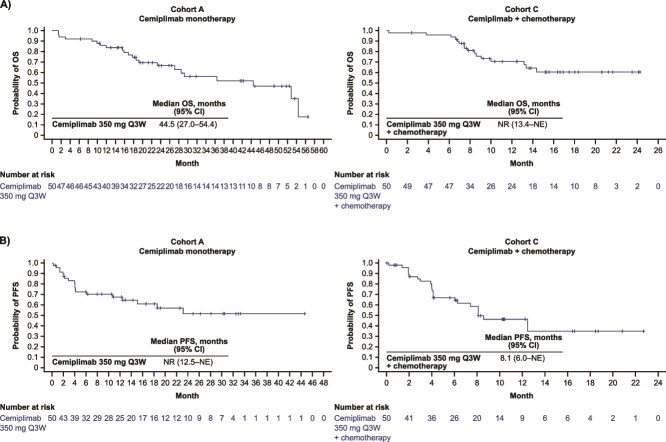
(A) OS and (B) PFS for cemiplimab monotherapy (Cohort A) and cemiplimab in combination with chemotherapy (Cohort C). Kaplan–Meier curves for OS and PFS were represented for cemiplimab monotherapy and cemiplimab + chemotherapy. Tick marks indicate censored observations. Data cut-off: 5 September 2023 (Cohort A). Data cut-off: 18 October 2023 (Cohort C). CI, confidence interval; NE, not evaluable, NR, not reached; PFS, progression-free survival; OS, overall survival; Q3W, every 3 weeks.

In Cohort A, median OS was 44.5 months (95% CI: 27.0–54.4; Fig. 2A Cohort A). At data cut-off, the probability of survival at 6 and 12 months was 92.0% (95% CI: 80.1–96.9) and 83.7% (95% CI: 70.1–91.5), respectively. Median PFS was not reached (95% CI: 12.5 months–NE; Fig. 2B Cohort A). Estimated PFS at 6 and 12 months was 72.6% (95% CI: 57.5–83.1) and 67.7% (95% CI: 52.2–79.2), respectively.

In Cohort C (cemiplimab + chemotherapy), the overall ORR was 42.0% (90% CI: 30.5–53.5%), with two CRs and 19 PRs. The observed DOR ranged from 2.3 to 20.7 months (Table 4). The majority of responding patients (76.2%) had an observed DOR >6 months. The Kaplan–Meier median DOR was not reached (6.6 months–NE). Clinical benefit was observed across all PD-L1 groups (Table 4). Patients with PD-L1 ≥50% (*n* = 14) had an ORR of 35.7%, those with PD-L1 1–49% (*n* = 16) had an ORR of 43.8%, and those with PD-L1 <1% (*n* = 11) had an ORR of 54.5%.

Median OS in Cohort C was not reached (95% CI: 13.4 months–NE; Fig. 2A Cohort C). At data cut-off, the estimated probability of survival at 6 and 12 months was 96.0% (95% CI: 84.8–99.0) and 70.6% (95% CI: 54.5–81.9), respectively. The median PFS was 8.1 months (95% CI: 6.0 months–NE; Fig. 2B Cohort C) and the estimated PFS at 6 and 12 months was 67.0% (95% CI: 51.3–78.6) and 46.4% (95% CI: 30.2–61.2), respectively.

### Immunogenicity

Of the 60 patients receiving cemiplimab monotherapy (Cohort A), 57 (95.0%) had immunogenicity assessments. Of the 50 patients receiving cemiplimab in combination with chemotherapy (Cohort C), 48 (96.0%) had immunogenicity assessments (Table 5).

**Table 5:** Summary of immunogenicity results for cemiplimab monotherapy or cemiplimab in combination with chemotherapy

***n* (%)**	**Cemiplimab monotherapy** **(Cohort A)** **(*n* = 57)**	**Cemiplimab + chemotherapy** **(Cohort C)** **(*n* = 48)**
ADA negative	50 (87.7)	39 (81.3)
Pre-existing immunoreactivity	2 (3.5)	6 (12.5)
ADA treatment-emergent response	5 (8.8)	3 (6.3)
Persistent	1 (1.8)	2 (4.2)
Transient	2 (3.5)	1 (2.1)
Indeterminate	2 (3.5)	0
NAb negative	57 (100.0)	47 (97.9)

ADA, anti-drug antibody; NAb, neutralizing antibody.

Treatment-emergent immunogenicity was low in both cohorts: observed in five (8.8%) patients receiving cemiplimab monotherapy and three (6.3%) patients receiving cemiplimab in combination with chemotherapy (Table 5). Overall, across both cohorts, patient ADA status did not result in reductions of cemiplimab exposure in serum.

## Discussion

Part 1 of this study confirmed that 350 mg cemiplimab monotherapy in Japanese patients has comparable safety and PK with the global population [5]. Part 2 assessed the safety, tolerability, PK, immunogenicity, and efficacy of cemiplimab as monotherapy or in combination with chemotherapy in Japanese patients with advanced NSCLC.

In patients who received cemiplimab monotherapy (Cohort A; *n* = 60), TEAEs of grade ≥3 occurred in 51.7% of patients, with no TEAEs that resulted in death. Pneumonitis was observed in 13 patients (21.7%) and three patients (5.0%) experienced a grade ≥3 event. Events of all grades were adequately managed by following treatment guidelines. No grade 5 pneumonitis events occurred. In Cohort A, TEAEs leading to treatment discontinuation were experienced by 22 (37.0%) patients, and grade ≥3 TEAEs leading to treatment discontinuation were experienced by 12 (20.0%). All patients recovered from these TEAEs at the time of data cut-off. Most of these patients (16/22) had a PR or CR, with a DOR range of 2.1–42.5 months. The proportion of patients with TEAEs that led to treatment discontinuation was higher in Study 1622 Part 2 Cohort A compared to EMPOWER-Lung 1. This may be in part attributable to small sample size as well as to a greater proportion of patients (40.0%) in the Study 1622 Part 2 Cohort A being exposed to cemiplimab for ≥48 weeks compared to only 23.7% of patients in EMPOWER-Lung 1.

In patients who received cemiplimab with chemotherapy (Cohort C; *n* = 50), TEAEs of grade ≥3 occurred in 68.0%, and two TEAEs resulted in a death (one case of immune-mediated pneumonitis, and one cerebellar hemorrhage unrelated to the study drug). The most common grade ≥3 TEAEs (in >5% of patients) were decreased neutrophil count, anemia, increased alanine aminotransferase, decreased WBC count, decreased appetite, neutropenia, and pneumonia, which are generally consistent with the known safety profile for cemiplimab in combination with chemotherapy. In EMPOWER-Lung 3, the most common grade ≥3 TEAEs with cemiplimab were anemia, neutropenia, and decreased WBC count [14]. In Cohort C, TEAEs leading to permanent treatment discontinuation were observed in 19 patients (38.0%). Seven patients discontinued treatment due to grade 1 and 2 events, and nine patients had recovered from these TEAEs at the time of database lock. A majority of the 19 patients derived benefit from cemiplimab treatment (11 patients had stable disease, and four patients had a PR). Although the proportion of patients who discontinued treatment due to TEAEs was higher in Cohort C than in EMPOWER-Lung 3 (5.1%) [14], this may also be attributable to the small sample size; importantly, it was not associated with any significant changes in the efficacy outcomes observed.

The TEAEs that led to discontinuation in this study were generally similar to those observed in pivotal studies [13, 14], including pneumonitis, hepatic abnormal function in patients treated with cemiplimab monotherapy and infusion-related reactions, skin reactions, and lab abnormalities (liver enzymes and creatinine elevation) in patients treated with cemiplimab + chemotherapy.

Overall, the safety results in this study were generally consistent with the established safety profiles of cemiplimab as monotherapy or in combination with chemotherapy from phase 3 pivotal studies [13, 14], as well as with the results from other studies of anti–PD-1 agents in Japanese patients [18–21].

There are no new important risks reported for the Japanese population that have not previously been observed in the rest of the world population for patients with NSCLC.

For the efficacy evaluation in patients receiving cemiplimab monotherapy (Cohort A), the response rate to cemiplimab monotherapy was 60.0% among patients with centrally confirmed PD-L1 ≥50%, with a greater response in those with higher PD-L1 expression levels (Table 4). This high response rate, along with a durable DOR (87% of patients with a DOR ≥6 months and 50% of patients with a DOR ≥12 months) and OS (median 44.5 months), support a clinically meaningful benefit of cemiplimab in Japanese patients with advanced NSCLC with PD-L1 expression levels ≥50%.

The efficacy observed in Cohort A was consistent with the EMPOWER-Lung 1 study, in which cemiplimab demonstrated a statistically significant and clinically meaningful survival benefit compared with platinum-based chemotherapy, as shown by OS (22.1 months vs. 14.3 months; hazard ratio [HR] = 0.68, *P* = 0.0022), PFS (6.2 months vs. 5.6 months; HR = 0.59, *P* < 0.0001), and ORR (36.5% vs. 20.6%; *P* < 0.0001) [13]. The findings from this study and EMPOWER-Lung 1 represent a clinically important advancement for the first-line treatment of Japanese patients with advanced NSCLC.

Similarly, in patients receiving cemiplimab in combination with chemotherapy (Cohort C), ORR was observed in 42.0%, with a clinical benefit observed across all PD-L1 levels. Small sample sizes across different PD-L1 expression groups limited the interpretation of the relationship between PD-L1 levels and efficacy. A durable DOR was observed, with 76.2% of patients having a DOR of ≥6 months. The median PFS was 8.1 months. Median OS had not been reached at data cut-off; at 6 months, 96.0% of patients were alive.

Efficacy in Cohort C was consistent with that of the EMPOWER-Lung 3 study, in which cemiplimab plus chemotherapy compared with placebo plus chemotherapy demonstrated a statistically significant and clinically meaningful greater median OS (21.9 months vs. 13.0 months; HR = 0.71, two-sided *P-*value 0.014), PFS (8.2 months vs. 5.0 months; HR = 0.556, *P* < 0.0001), and ORR (43.3% vs. 22.7%, *P* < 0.0001) [14]. Overall, these results support the conclusion that cemiplimab in combination with chemotherapy provided a clinically meaningful benefit in Japanese patients.

In this study, PK parameters after the first dose and at steady state in Cohort A were as expected. For Cohort C, PK parameters were similar to Cohort A, further demonstrating no effect of chemotherapy on cemiplimab exposure. The PK results for Cohort A (Japanese patients) were similar to non-Japanese patients in EMPOWER-Lung 1. In non-Japanese patients, mean (SD) C_trough_ was 22.1 (14.8) mg/L and C_max_ was 120 (65.0) mg/L following the first dose; mean (SD) C_trough_ was 61.7 (29.4) mg/L and C_max_ 181 (77.3) mg/L at steady state [22]. In Cohort A, a slightly higher mean exposure of cemiplimab was reported following the first dose (mean [SD] C_trough:_ 26.6 [7.67] mg/L; C_max_ 145 [37.8] mg/L) and at steady state (mean [SD] C_trough_ 67.8 [23.5] mg/L; C_max_ 193 [36.9] mg/L). This may be attributed to the lower mean body weight of 58.8 kg observed in Cohort A versus 70.8 kg in EMPOWER-Lung 1.

Similar PK results were reported for Cohort C when compared with patients (non-Japanese) of EMPOWER-Lung 3. In non-Japanese patients, mean (SD) C_trough_ was 21.5 (18.4) and C_max_ was 95.3 (48.6) mg/L following the first dose; and mean (SD) C_trough_ was 48.6 (25.0) and C_max_ was 129 (46.9) mg/L at steady state [22]. In Cohort C, a higher mean exposure of cemiplimab was reported following the first dose (mean [SD] C_trough_ 25.1 [14.3] mg/L; C_max_ 144 [36.9] mg/L) and at steady state (mean [SD] C_trough_ 81.6 [45.1] mg/L; C_max_ 181 [40.8] mg/L), which again may be a result of a lower mean body weight (60.0 kg in Cohort C vs. 73.4 kg in EMPOWER-Lung 3).

The administration of monoclonal antibody therapies may induce humoral immune responses that lead to the formation of ADAs. High levels of ADAs may lead to altered PK, reduced efficacy, or an impact on safety [23]. The incidence of treatment-emergent ADAs was low in both cohorts in this study (5/57; 8.8% in Cohort A and 3/48; 6.3% in Cohort C), as well as in EMPOWER-Lung 1 (2/162; 1.2%) [22] and EMPOWER-Lung 3 (7/200; 3.5%) [14]. In addition, the incidence of treatment-emergent ADAs did not appear to influence cemiplimab concentrations in the serum. These results are consistent with the low immunogenicity of cemiplimab observed across various studies and tumor types [24].

This study was designed as a single-arm study in Japanese patients only. The study showed consistency between Japanese patients and the controlled global studies of non-Japanese patients with advanced NSCLC treated with cemiplimab with or without chemotherapy. Cemiplimab is currently one of two immune checkpoint inhibitors (along with pembrolizumab) that has demonstrated a survival benefit as monotherapy and in combination with chemotherapy as the first-line treatment in patients with advanced NSCLC with both the squamous and non-squamous histology [13, 14, 16, 25]. Available data from cemiplimab studies showed long-term survival benefits at 5-year follow-up, favorable patient-reported outcomes, and clinical benefits in patients with both squamous and non-squamous histologies and also those with locally advanced NSCLC who were not candidates for concurrent chemoradiation [16, 25–29]. Together these data support cemiplimab as an additional therapeutic option available to Japanese patients with advanced NSCLC for physicians’ consideration when they determine treatment plans based on each individual patient’s needs and conditions.

In conclusion, cemiplimab, both as monotherapy and in combination with chemotherapy, demonstrated efficacy in Japanese patients with advanced NSCLC. Safety was generally consistent with the known safety profile of cemiplimab and of chemotherapy. Immunogenicity was low in both cohorts, and ADA status did not result in reductions in cemiplimab exposure in serum. Overall, cemiplimab demonstrated a favorable benefit–risk profile in Japanese patients with squamous or non-squamous advanced NSCLC with no *EGFR*, *ALK*, or *ROS1* aberrations.

## Abbreviations

ADA, anti-drug antibody; *ALK,* anaplastic lymphoma kinase; CI, confidence interval; C_max,_ maximum concentration; CR, complete response; C_trough,_ trough concentration; DOR, duration of response; ECOG, Eastern Cooperative Oncology Group; *EGFR,* epidermal growth factor receptor; HR, hazard ratio; IHC, immunohistochemistry; IRC, independent review committee; IV, intravenous; NE, not evaluable; NSCLC, non-small cell lung cancer; ORR, objective response rate; OS, overall survival; PD-1, programmed cell death-1; PD-L1, programmed cell death-ligand 1; PFS, progression-free survival; PK, pharmacokinetics; PR, partial response; Q3W, every 3 weeks; RECIST 1.1, Response Evaluation Criteria in Solid Tumors version 1.1; *ROS1,* ROS proto-oncogene 1; SD, standard deviation; TEAE, treatment-emergent adverse event; WBC, white blood cell.

## Supplementary Material

Sato_Japan_study_1622_MAN_Suppl_19Sept2025_hyaf160

## Data Availability

Qualified researchers may request access to study documents (including the clinical study report, study protocol with any amendments, blank case report form, and statistical analysis plan) that support the methods and findings reported in this manuscript. Individual anonymized participant data will be considered for sharing: (i) once the product and indication has been approved by major health authorities (e.g. FDA, EMA, PMDA, etc.) or development of the product has been discontinued globally for all indications on or after April 2020 and there are no plans for future development, (ii) if there is legal authority to share the data, and (iii) there is not a reasonable likelihood of participant re-identification. Submit requests to https://vivli.org/.
