# Supplementary material for: Cemiplimab in Japanese patients with advanced non-small cell lung cancer
Source: Jpn J Clin Oncol. 2025 Oct 29;56(1):55–65. doi: 10.1093/jjco/hyaf160 (PMC12784360; doi:10.1093/jjco/hyaf160)
Supplement: Sato_Japan_study_1622_MAN_Suppl_19Sept2025_hyaf160 [file sato_japan_study_1622_man_suppl_19sept2025_hyaf160.docx]

Supplementary Table 1. Summary of treatment-related TEAEs for cemiplimab monotherapy or cemiplimab in combination with chemotherapy

| ***n* (%)** | **Any grade** | **Grade ≥3** |
| --- | --- | --- |
| Cemiplimab monotherapy (Cohort A) (*n*= 60) |  |  |
| Treatment-related TEAEs |  |  |
| Any | 55 (91.7) | 18 (30.0) |
| Leading to treatment discontinuation | 19 (31.7) | 10 (16.7) |
| Leading to death | 0 | 0 |
| Treatment-related TEAEs occurring in ≥10% of patients (any grade arm) |  |  |
| Infusion-related reaction | 19 (31.7) | 1 (1.7) |
| Pruritus | 14 (23.3) | 0 |
| Pneumonitis | 12 (20.0) | 3 (5.0) |
| Rash | 10 (16.7) | 0 |
| Hypothyroidism | 9 (15.0) | 0 |
| Malaise | 7 (11.7) | 0 |
| Dry skin | 6 (10.0) | 1 (1.7) |
| Diarrhea | 6 (10.0) | 1 (1.7) |
| Nausea | 6 (10.0) | 0 |
| Cemiplimab + chemotherapy (Cohort C) (*n* = 50) | | |
| Treatment-related TEAEs |  |  |
| Any | 50 (100.0) | 32 (64.0) |
| Leading to treatment discontinuation | 19 (38.0) | 12 (24.0) |
| Leading to death | 1 (2.0) | NR |
| Treatment-related TEAEs occurring in ≥10% of patients (any grade arm) | **Any grade  *n* (%)** | **Grade ≥3  *n* (%)** |
| Anemia | 20 (40.0) | 5 (10.0) |
| Constipation | 15 (30.0) | 0 |
| Decreased appetite | 13 (26.0) | 3 (6.0) |
| Nausea | 13 (26.0) | 1 (2.0) |
| Neutrophil count decreased | 13 (26.0) | 10 (20.0) |
| Alopecia | 11 (22.0) | 0 |
| Malaise | 10 (20.0) | 0 |
| Platelet count decreased | 9 (18.0) | 2 (4.0) |
| Arthralgia | 8 (16.0) | 0 |
| Peripheral sensory neuropathy | 8 (16.0) | 0 |
| Diarrhea | 7 (14.0) | 2 (4.0) |
| Rash | 7 (14.0) | 0 |
| Stomatitis | 7 (14.0) | 0 |
| Infusion-related reaction | 6 (12.0) | 2 (4.0) |
| Rash maculo-papular | 6 (12.0) | 0 |
| White blood cell count decreased | 6 (12.0) | 3 (6.0) |
| Eczema | 5 (10.0) | 0 |
| Edema peripheral | 5 (10.0) | 0 |
| Pneumonitis | 5 (10.0) | 1 (2.0) |
| Pruritus | 5 (10.0) | 1 (2.0) |

Adverse events were graded according to the National Cancer Institute Common Terminology Criteria for Adverse Events version 4.03.

NR, not reported; TEAE, treatment-emergent adverse event.

Supplementary Table 2. Summary of sponsor identified immune-related TEAEs results for cemiplimab monotherapy or cemiplimab in combination with chemotherapy

| ***n* (%)** | **Any grade** | **Grade ≥3** |
| --- | --- | --- |
| Cemiplimab monotherapy (Cohort A) (*n*= 60) |  |  |
| Immune-mediated TEAEs (sponsor-identified) |  |  |
| Any | 24 (40.0) | 8 (13.3) |
| Treatment-related TEAEs occurring in ≥1.7% of patients (any grade arm) |  |  |
| Hypothyroidism | 9 (15.0) | 0 |
| Pneumonitis | 8 (13.3) | 3 (5.0) |
| Adrenal insufficiency | 1 (1.7) | 0 |
| Arthralgia | 1 (1.7) | 0 |
| Diabetes mellitus | 1 (1.7) | 1 (1.7) |
| Encephalitis | 1 (1.7) | 1 (1.7) |
| Enterocolitis | 1 (1.7) | 0 |
| Hepatic function abnormal | 1 (1.7) | 1 (1.7) |
| Hyperthyroidism | 1 (1.7) | 0 |
| Hypopituitarism | 1 (1.7) | 0 |
| Rash | 1 (1.7) | 0 |
| Rash maculo-papular | 1 (1.7) | 1 (1.7) |
| Thyroiditis | 1 (1.7) | 0 |
| Type 1 diabetes mellitus | 1 (1.7) | 1 (1.7) |
| Cemiplimab + chemotherapy (Cohort C) (*n* = 50) | | |
| Immune-mediated TEAEs (sponsor-identified) |  |  |
| Any | 18 (36.0) | 9 (18.0) |
| Treatment-related TEAEs occurring in ≥2% of patients (any grade arm) |  |  |
| Adrenal insufficiency | 4 (8.0) | 1 (2.0) |
| Pneumonitis | 4 (8.0) | 1 (2.0) |
| Erythema multiforme | 2 (4.0) | 2 (4.0) |
| Hypothyroidism | 2 (4.0) | 0 |
| Alanine aminotransferase increased | 1 (2.0) | 1 (2.0) |
| Aspartate aminotransferase increased | 1 (2.0) | 1 (2.0) |
| Colitis | 1 (2.0) | 1 (2.0) |
| Encephalitis | 1 (2.0) | 1 (2.0) |
| Hyperthyroidism | 1 (2.0) | 0 |
| Interstitial lung disease | 1 (2.0) | 1 (2.0) |
| Pruritus | 1 (2.0) | 1 (2.0) |
| Rash | 1 (2.0) | 0 |
| Rash maculo-papular | 1 (2.0) | 0 |
| Tubulointerstitial nephritis | 1 (2.0) | 1 (2.0) |

TEAE, treatment-emergent adverse event.
